# Supplementary material for: Atomic faulting drives exceptional toughness in low thermal expansion chromium alloys
Source: Nat Commun. 2026 Feb 6;17:2435. doi: 10.1038/s41467-026-69365-5 (PMC12988168; doi:10.1038/s41467-026-69365-5)
Supplement: Supplementary file 1 — Supplementary Information [file 41467_2026_69365_MOESM1_ESM.pdf]

Supplementary Information

Atomic faulting drives exceptional toughness in low thermal expansion chromium alloys

Chengyi Yu *et al*

Supplementary Note 1

Supplementary Discussion 1 | The physical origin of ZTE of chromium-based alloy.

The Cr-Fe-Ge system was selected based on preliminary phase stability considerations. Initial doping studies on Cr-Fe binary alloys revealed that the $Cr_{0.96}Fe_{0.04}$ composition exhibited the most pronounced negative thermal expansion (NTE) (Supplementary Figure 1a). To modulate the thermal expansion from negative to near-zero, we introduced elements (e.g., Cu, Al, Ge) known to influence electron density and magnetic exchange interactions. Among these, Ge demonstrated optimal tunability of the thermal expansion coefficient (TEC) across a systematic composition range (x = 0 - 2.5 *at.*%, Supplementary Figure 1b-d).

Supplementary Discussion 2 | The physical origin of ZTE of chromium-based alloy.

The anomalous thermal expansion of metal materials is usually considered to be related to the magneto-volume effect, that is, spin-lattice coupling [1, 2]. Macroscopic magnetic measurements demonstrate an antiferromagnetic-paramagnetic transition (T_N =315 K) in $CFGB_0$ (Supplementary Figure 3a-b). Consistent with the zero thermal expansion behavior, this transition indicates that the anomalous thermal expansion may stem from magnetic contributions. The results of the variable temperature neutron diffraction spectrum show that $CFGB_0$ exhibits antiferromagnetic ordering at low temperatures, and the atomic magnetic moment has been extracted (Supplementary Figure 3c-d). In magnetic materials, lattice thermal expansion (ω_{exp}) is usually composed of spontaneous magnetostriction (ω_s) caused by magnetic contribution and normal harmonic thermal vibration caused by phonon contribution (ω_{nm}) :

$$\omega_s = \omega_{exp} - \omega_{nm} \quad (4)$$

where the ω_{nm} is the nominal thermal expansion calculated based on the Debye-Grüneisen model (which describes the intrinsic lattice thermal expansion due to thermal vibration). Supplementary Figure 4a shows the relationship between ω_s and ω_{nm} of $CFGB_0$ (S-3), the thermal contraction caused by magnetic contribution (ω_s) is equal to the lattice expansion due to thermal vibration (ω_{nm}) below the T_N induced to ZTE. To further study the evolution details of the magnetic order and the magnetic contribution to the lattice (ω_s), the ω_s versus $M(Cr/Fe)^2$ were studied under the guidance of Ginzburg–Landau theory, the ω_s are described according to the following formula (5):

$$\omega_s = \kappa C M^2 \qquad (5)$$

where κ represents the compressibility and C is the magneto-volume coupling constant; M is the magnetic moment related to the thermal expansion. As shown in Supplementary Figure 4b, ω_s versus $M(Cr/Fe)^2$ have a linear relationship in the range of zero thermal expansion, suggesting the ferromagnetic order is key to controlling the ZTE behavior.

Supplementary Discussion 3 | Effect of Cr_2B precipitation on thermal expansion.

We synthesized a single-phase polycrystalline compound of Cr_2B. XRD confirmed that the sample was an orthorhombic (Space group: *Fddd*) crystal structure without impurity (Supplementary Figure 7a). The dilatometer thermal expansion shows positive thermal expansion (PTE, $\alpha_l = 6.53 \times 10^{-6}$ K^{-1}, 200 - 315 K, Supplementary Figure 7b), which is not large compared to traditional metals (α-Fe, $\alpha_l = 14.0 \times 10^{-6}$ K^{-1}). Thus, the lower content has little effect on the increase in thermal expansion.

To accurately evaluate the lattice thermal expansion behavior of $CFGB_1$ alloy, we performed variable temperature synchrotron X-ray diffraction (Supplementary Figure 7c-d). The lattice thermal expansion of the dual-phase alloy was calculated as follows (6) and (7):

$$\alpha_l = \frac{(\Sigma a_1 - \Sigma a_0)}{3\Sigma a_0} / (T_0 - T_0) \qquad (6)$$

$$\Sigma a = mol._{Cr2B}\% \times V_{Cr2B} + mol._{BCC}\% \times V_{BCC} \qquad (7)$$

where α_l is the apparent lattice thermal expansion; $mol._{Cr2B}\%$ and $mol._{BCC}\%$ are molar fractions of the Cr_2B and BCC phase determined by the results of SXRD data.

As shown in Supplementary Figure 7e, the lattice thermal expansions are $\alpha_l = 5.4 \times 10^{-6}$ K^{-1} for the Cr_2B phase and $\alpha_l = 51.8 \times 10^{-6}$ K^{-1} for the BCC phase. The apparent lattice thermal

expansion of the $CFGB_1$ alloy is $\overline{\alpha}_l \approx 2.2 \times 10^{-6}$ K^{-1} (200 - 315 K), which corroborates to dilatometer measurement.

Supplementary Discussion 4 | Toughness calculation and corrosion resistance.

The compression stress-strain curves at room temperature were measured on a CMT4105 universal electronic compressive testing machine using a Φ 5×8 mm cylinder and an initial strain rate of 0.25 mm/min (Supplementary Figure 8a), performed three times for each sample (Supplementary Figure 5). Unlike traditional structural materials, most ZTE/LTE alloys are very brittle and have almost no ductility. Therefore, we use the integral of the stress-strain curve to approximate its toughness (Supplementary Figure 8b).

It is worth mentioning that the chromium-based alloy has superior corrosion resistance to the currently commercial Invar alloy (Supplementary Figure 8c-e). There is no obvious change in soaking 3.5 *wt.* % NaCl solution for over 190 days, which is not his limit due to time constraints. This is due to it forming a dense chromium oxide (Cr_2O_3) passivation film on the metal surface. The passivation film is very stable and can effectively prevent the metal from further reacting with oxygen and moisture, thereby protecting the metal from corrosion. As a result, chromium-based LTE alloy can make up for the shortcomings of Invar alloy in extreme service environments (Supplementary Figure 8).

Supplementary Discussion 5 | Determination of phase structure of the $CFGB_1$ and the contribution of grain refinement.

The electro-probe micro-analyzer (EPMA) analyses (Supplementary Figure 10a-g) show that the introduction of boron atoms will segregate at the grain boundaries of the matrix (Cr-Fe-Ge, BCC) to form a second-phase precipitation. The selected area electron diffraction (SAED) under different crystal band axes by transmission electron microscopy (TEM, Supplementary Figure 10h-k) further confirmed that the precipitated phase may be the Cr_2B phase with the *Fddd* space group. However, it is still not confirmed that there is only one structure of the precipitate phase. As a result, the synchrotron X-ray diffraction (SXRD) profile was further refined by using the dual phases, which further verified that the second precipitate phase is Cr_2B (Figure 2a).

The microstructure is further revealed by the electron backscattered diffraction (EBSD, Supplementary Figure 11). In addition, we noticed that with the introduction of boron atoms, the

grain size of the CFGB$_x$ series alloys was significantly refined: 190 μm, 91 μm, and 25 μm for B_0, $B_{0.5}$, and B_1, respectively. This may be due to the precipitation of Cr_2B at the grain boundaries, preventing the further growth of the grains. To clarify the contribution of grain refinement (σ_{gs}) to the yield strength improvement, we estimate the *Hall-Petch* effect as follows (8):

$$\sigma_{gs} = K^{H\text{-}P} d^{-1/2} \qquad (8)$$

Here, *K* is the coefficient of *Hall-Petch,* and *d* is the grain size. It is noted that the value of *K* is approximately calculated using pure chromium metal 800 MPa · $\mu m^{1/2}$ [3]. Based on the calculation results (Supplementary Figure 12), we can conclude that the yield strength is mainly enhanced by the grain refinement effect of the series alloys.

Supplementary Discussion 6 | Confirmation of boron atom enrichment behavior at the interface.

Based on combined SXRD, EPMA, and EBSD analyses, the phase boundaries between the BCC matrix and Cr_2B phases are characterized as follows: (i) the two phases exhibit markedly different crystal structures with no coherent or semi-coherent relationship; (ii) the two phases exhibit random crystallographic orientations with no defined interface relationship; (iii) Cr_2B predominantly precipitates at grain boundaries, accounting for over 90% of all interfacial connections. This raises a critical question: given the large differences in orientation and crystal structure between the BCC/Cr_2B phase interface, why does the sample exhibit improved plasticity instead of failing due to interfacial brittleness during deformation? Although stacking fault deformation within the Cr_2B phase can release some interfacial stress, a stable interfacial structure is indispensable.

Therefore, we employed atom probe tomography (APT) to analyze the phase interface structure between BCC and Cr_2B (Fig. 2d-h). A region of interest (yellow cube) was selected at the interface to facilitate detailed analysis of the elemental distribution (Fig. 2e and f). The spatial distribution map of boron atoms clearly shows their enrichment at the phase boundary (Fig. 2g, red spheres represent B atoms). Notably, the phase boundary (dashed line) was objectively determined based on elemental distribution mapping.

To quantify the boron enrichment at the interface more objectively, we further analyzed the Gibbs interfacial excess of the interface. Specifically, we selected the boron 7-*at.*% iso-concentration surface to calculate the proximity histograms (Supplementary Figure 14a and b), which corresponds to a more appropriate interface position (Supplementary Figure 14c). Indeed, B is enriched between the BCC/Cr_2B boundary of 4.17 ± 0.26 nm^{-2}, while Cr is considerably depleted between -0.93 ± 0.26 nm^{-2}. Fe and Ge exhibit no enrichment or depletion of -0.04 ± 0.01 nm^{-2} and 0.15 ± 0.00 nm^{-2}, as shown in Supplementary Figure 14d-g. This further clarifies the behavior of boron atoms enriching at grain boundaries.

Supplementary Discussion 7 | In-situ loading neutron diffraction measurements.

An in-situ loading neutron diffraction study by neutron diffraction was performed at the VULCAN beamline (BL-7) in Oak Ridge National Laboratory (ORNL), USA. The analysis of neutron data was based on VDRIVE software and GASA software. As shown in Supplementary Figure 16a, the sample is placed in the middle of the detector at a Ω = 45 degrees. While applying axial compressive stress (Supplementary Figure 16b), bank 1 and bank 2 collect diffraction patterns along the loading direction (LD) and transverse direction (TD), respectively (Supplementary Figure 16c-h).

The analysis of neutron data was based on VDRIVE software and GASA software. The single peak fitting method was used to determine the lattice strain of the specific (*h k l*) reflections during loading. The lattice strain was calculated using the formula (9):

$$\text{Strain} = \frac{(d_1 - d_0)}{d_0} \times 100\% \qquad (9)$$

Here, d_1 and d_0 represent the interplanar crystal spacing of the (*h k l*) crystal plane after and before loading, respectively. For average lattice strain (ε_i), the d_1 and d_0 are replaced by the unit cell parameters (a_1 and a_0). The phase-specific stress was calculated by the following formula (10):

$$\sigma_i = \frac{E_i}{(1+v_i)(1-2v_i)} \times \{(1 - v_i) \times \varepsilon_{i,11} + v_i \times (\varepsilon_{i,22} + \varepsilon_{i,33})\} + \sigma_r \qquad (10)$$

Where *i* stands for BCC and Cr_2B, σ_i is the stress in the loading direction, E_i is the diffraction elastic modulus, v_i is Poisson's ratio, σ_r is the thermal residual stress, $\varepsilon_{i,11}$ is the lattice strain in LD, $\varepsilon_{i,22}$ and $\varepsilon_{i,33}$ are the lattice strains in TD and ND, respectively. The $\varepsilon_{i,22} = \varepsilon_{i,33}$ can be measured by TD.

The modified *Williamson-Hall* (MWH) method was used to calculate dislocation density, which quantifies the effects of average sub-grain (crystallite) size D and micro-strain fields on line broadening as formulas (11) and (12):

$$(\Delta K)^2 = (0.9/D)^2 + (\pi A^2 b^2/2\rho) \times (K^2\bar{C}) + O(K^2\bar{C})^2 \qquad (11)$$

$$\bar{C} = \overline{C}(1-qH^2) \qquad (12)$$

Here, $K = 1/d$, $\Delta K = -K(\Delta d/d)$, d is the interplanar spacing, Δd is FWHM, b is the Burgers vector length of the dislocation, ρ is dislocation density, A is a constant that depends on the effective outer cutoff radius of dislocations, besides $O(K^2\bar{C})$ represents the non-interpreted high-order terms. $\bar{C}$ is the average dislocation contrast factor.

Supplementary Discussion 8 | Determination of stacking fault behavior.

The results of ex-situ transmission electron microscopy (TEM) show that stacking faults are observed in the precipitated phases under different strains (ε = 2.5, ε = 7.5 %, and ε = 15 %), as shown in Supplementary Figure 20a-c. Even if random grains with different orientations are selected, the orientation of stacking faults is the {001} crystal plane family, which can be confirmed by selecting the diffraction traces of electron diffraction (Insert in Supplementary Figure 20a-c). This indicates that the deformation of Cr_2B has strong anisotropy, which is why it can both coordinate deformation and improve the work-hardening ability of the alloy.

In addition, we selected a single stacking fault and conducted an atomic-scale analysis (Supplementary Figure 21). The results of the HAADF image show that stacking faults are preferentially activated on the boron atomic plane under low strain (ε = 2.5 %). Even after large plastic deformation, stacking fault slip is still only observed on the boron atomic plane. Combined with the crystal structure of Cr_2B, this can also indirectly confirm the possibility of a layered structure for improving mechanical properties.

Supplementary Note 2

Supplementary Table 1 | The toughness in typical ZTE materials. Noted： Rare earth-based compounds and their alloys are very active and not resistant to corrosion (3.5 *wt.* % NaCl solution). Invar is a completely plastic alloy, so for comparison, we can choose a strain of $\varepsilon = 30$ %. And the corrosion resistance of CFGB alloy far exceeds 190 days, but the limit was not measured due to time constraints.

Typical ZTE materials		Toughness ($J \cdot cm^{-3}$)	Corrosion resistance (days)	References
Re-based intermetallics	$Ho_2Fe_{16}Co$	0.72	0	[4]
	$Er_2Fe_{14}B$	1.2	0	[5]
	$LaFe_{11}Si_2H_x$	1.46	0	[6]
	GDFC	1.6	0	[7]
	$Tb(Co, Fe)_2$	2.96	0	[8]
Laves phase	$Hf_{0.87}Ta_{0.13}Fe_2$	2.9	~14	[9, 10]
Invar	$Fe_{65}Ni_{35}$	134.7	21	$\varepsilon = 30\%$
Mn_3Ge	Mn_3Ge	7.98	0	[11]
Dual-phase alloys	$La(Fe, Si)_{13}/Resin_3$	3.81	0	[12]
	La-Fe-Co-Si	277.8	0	[13]
	Er-Fe-V-Mo	9.55	0	[14]
	$LaFe_{18.2}Co_{1.2}Si_{1.6}$	11.8	0	[12]
	Er-Fe-B	16.88	0	[5]
	La(Fe,Co,Si)/Cu	34.93	0	[15]
	$Ho_{0.04}Fe_{0.96}$	106.8	0	[16]
$CFGB_x$ series	CFGB	46	＞190	This work
	$CFGB_{0.5}$	157.1	＞190	This work
	$CFGB_1$	252.1	＞190	This work

Supplementary Table 2 | The summary of the chemical bonds of the Cr-B within the A-B layer and the Cr-Cr bonds with the B-C layer.

Types of chemical bonds	Bond length (Å)	Orbital interaction	ICOHP
B1-Cr30	2.23136	2*p*-3*d*	0.83276
B2-Cr30	2.22605	2*p*-3*d*	0.82387
B3-Cr30	2.23135	2*p*-3*d*	0.83477
B4-Cr30	2.22603	2*p*-3*d*	0.83253
Cr30-Cr2	2.75733	3*d*-3*d*	0.32729
Cr30-Cr4	2.78787	3*d*-3*d*	0.32729
Cr30-Cr14	2.78785	3*d*-3*d*	0.27829
Cr30-Cr16	2.75733	3*d*-3*d*	0.31146
Cr32-Cr2	2.78784	3*d*-3*d*	0.27845
Cr30-Cr4	2.75732	3*d*-3*d*	0.31193
Cr30-Cr18	2.4722	3*d*-3*d*	0.86645
Cr30-Cr20	2.47222	3*d*-3*d*	0.86658

Supplementary Note 3

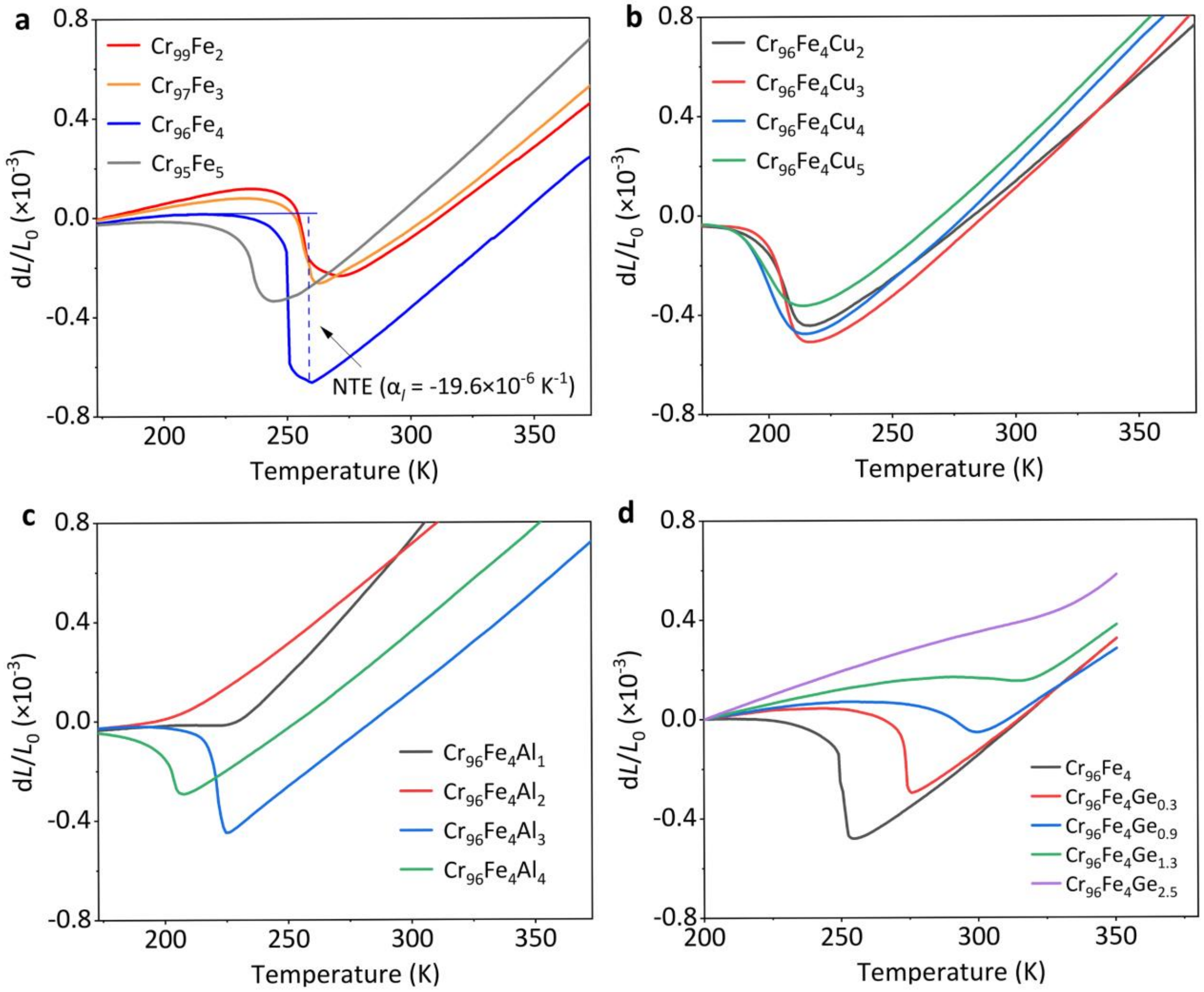

Supplementary Figure 1 | The dilatometer thermal expansion of the series alloys. a, the dilatometer thermal expansion of the Cr-Fe binary alloys. **b-d,** the chemical modulation of the Cr-Fe-X (X = Cu (**b**), Al (**c**), and Ge (**d**)) ternary alloys.

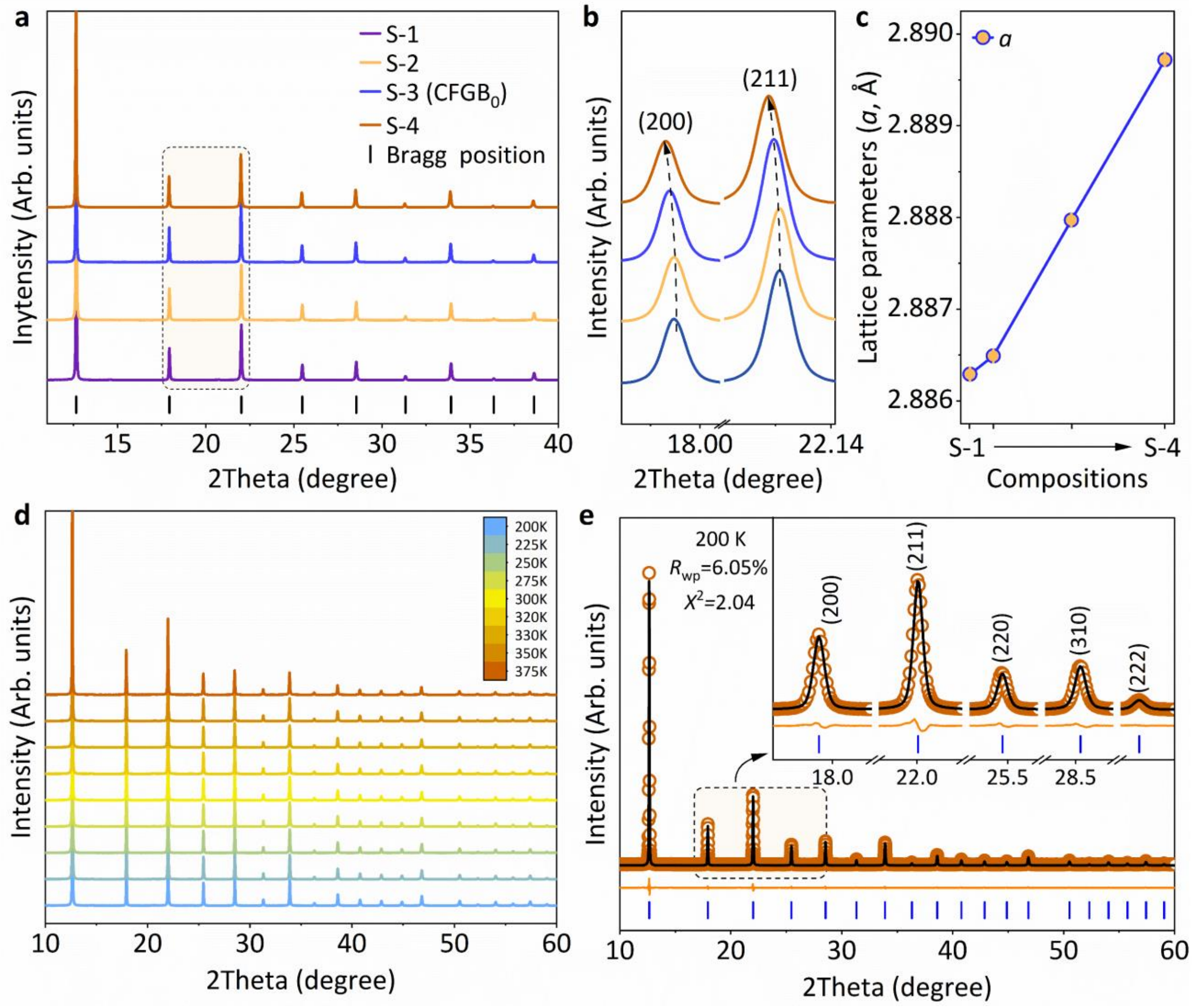

Supplementary Figure 2 | Crystal structure and lattice thermal expansion. a-b, Synchrotron X-ray diffraction (SXRD) profiles for the $Cr_{96}Fe_4Ge_x$ (x = 0, 0.3, 1.3 and 2.5, *at.* %, labeled as S-1, S-2, S-3 ($CFGB_0$) and S-4 respectively). **c,** The Lattice parameter of the series alloys. Error bars represent standard deviation. **d**, Temperature-dependent temperature SXRD profiles of the S-3 alloy. **e,** Rietveld refinement of SXRD profile at 200 K.

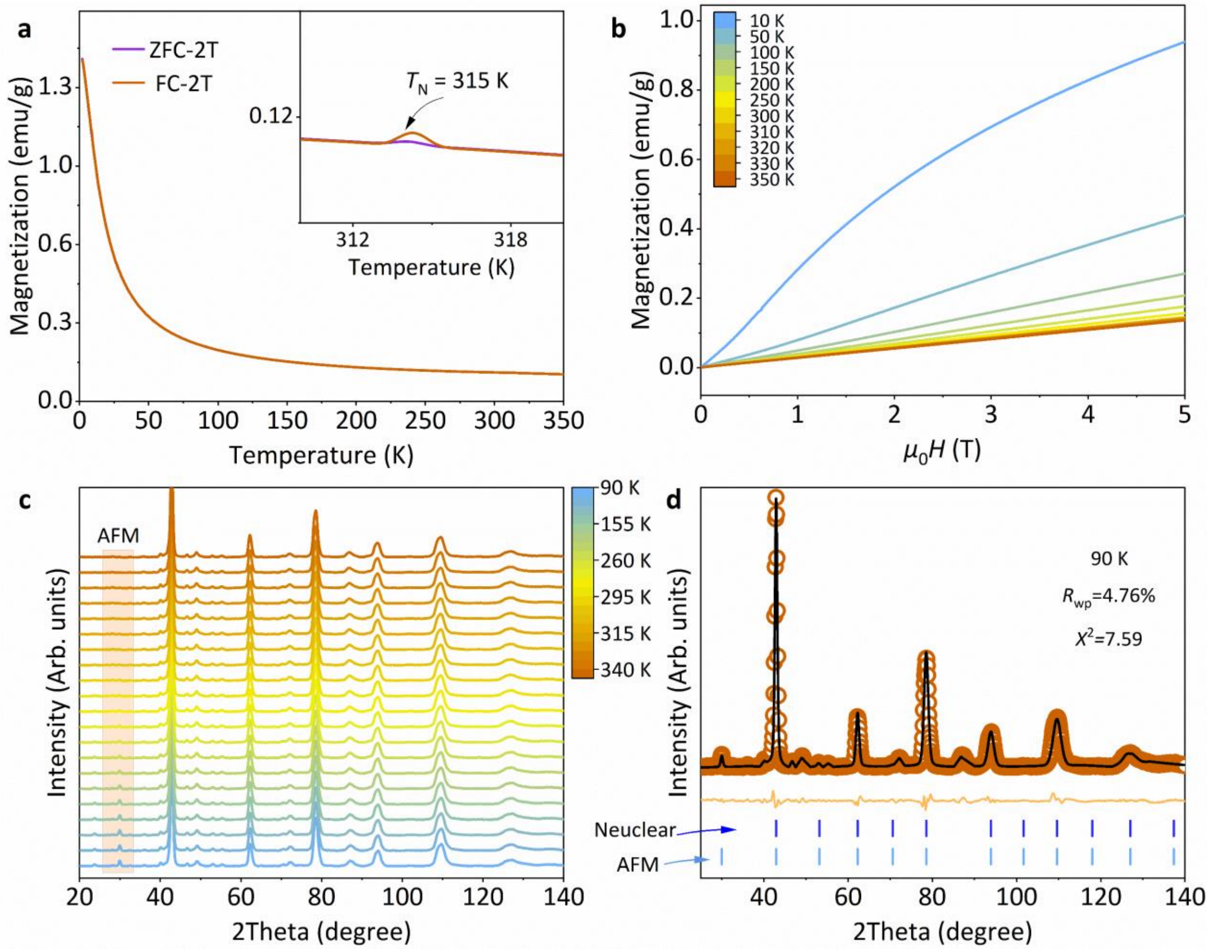

Supplementary Figure 3 | Macroscopic magnetic measurements and neutron powder diffraction profiles. a, Field-cooling (FC) and zero field-cooling (ZFC) magnetization (*M*) of S-3 alloy under 2 T magnetic field. **b,** Temperature dependence of magnetization (*M*−*T*) in an applied magnetic field for the S-3 alloy. **c**, NPD profiles of the S-3 alloy at various temperatures. **d,** Rietveld refinement of NPD profile at 90 K.

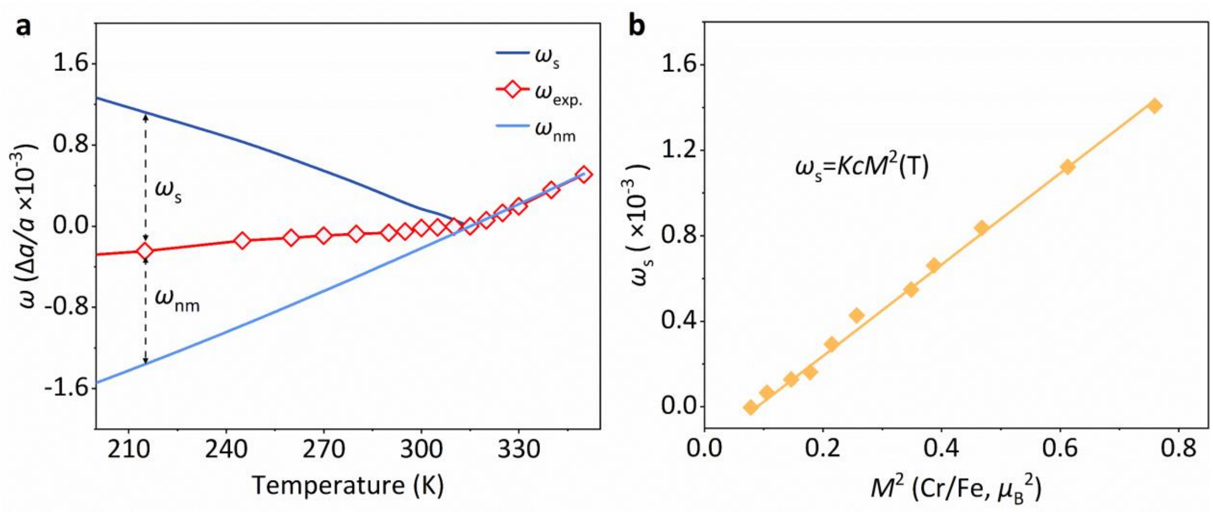

Supplementary Figure 4 | The origin of ZTE behavior. a, Comparison of ω_s and ω_{nm} corresponding to S-3. **b**, ω_s of S-3 as a function of the square of the $M_{(Cr/Fe)}$.

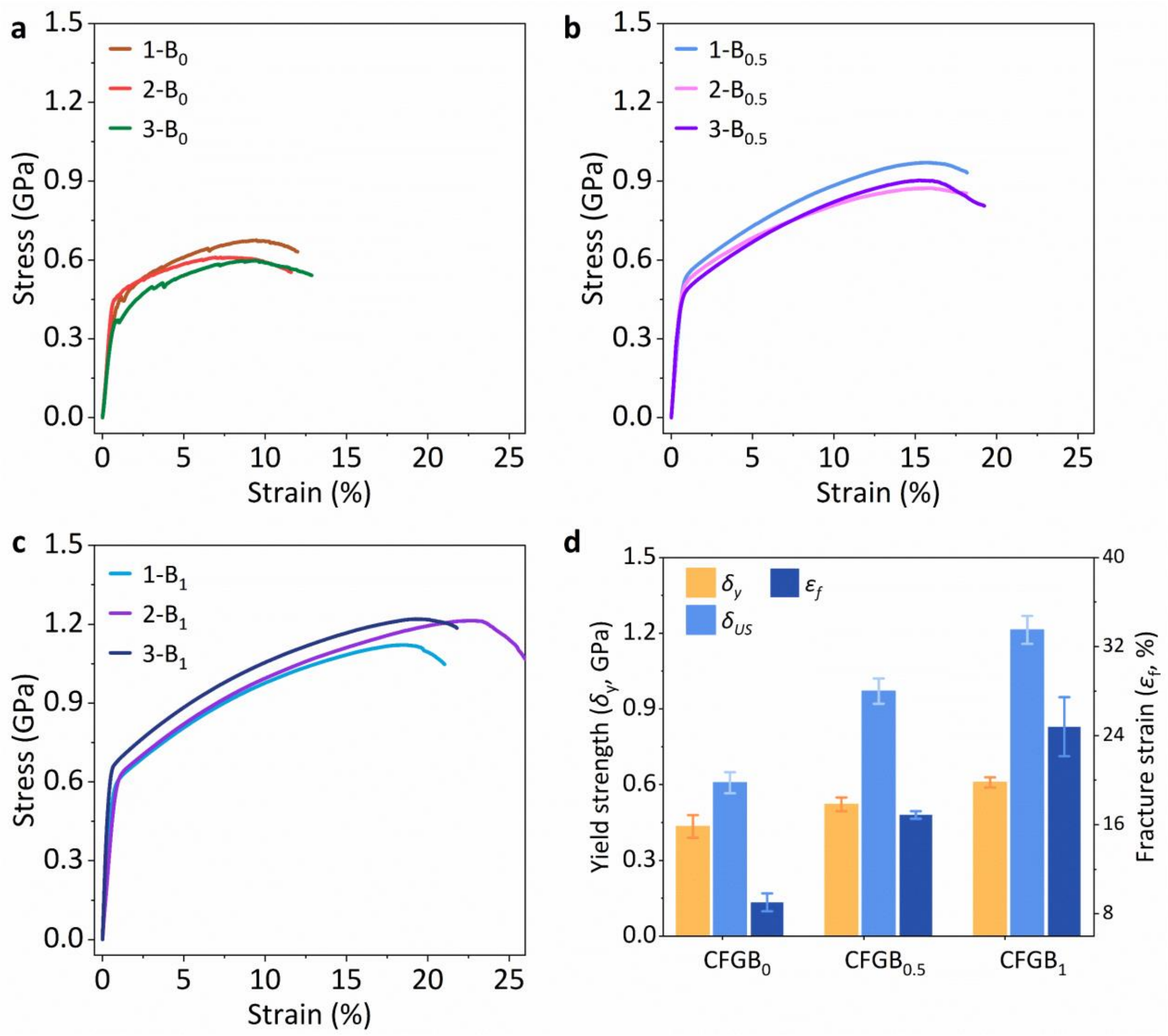

Supplementary Figure 5 | The mechanical properties of the series alloys. a-c, The stress-strain curves of CFGB$_x$ from multiple tests. **d**, The yield strength (σ_{y}), compressive strength (σ_{US}) and fracture strain (ε_{f}), respectively. Error bars represent standard deviation.

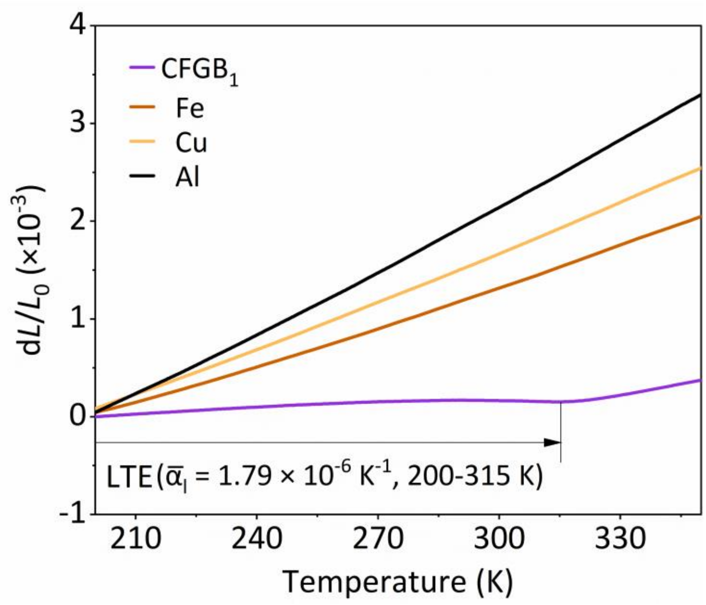

Supplementary Figure 6 | Comparison of dilatometer thermal expansion of GFGB1 and conventional alloys.

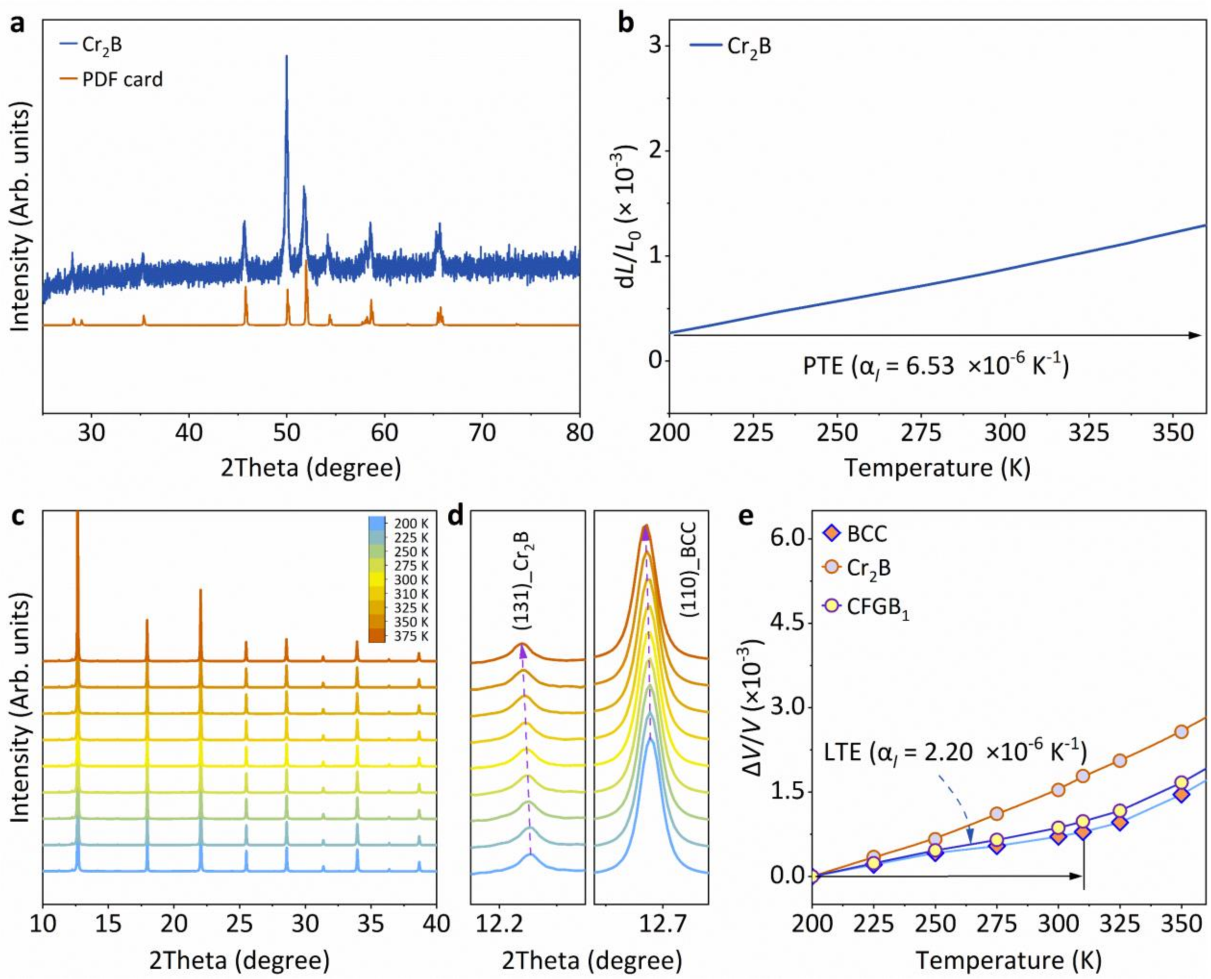

Supplementary Figure 7 | Lattice thermal expansion of the $CFGB_1$ alloy. a, XRD profiles of the targeted Cr_2B alloy. **b**, Dilatometer thermal expansion of the Cr_2B alloy. **c-d**, SXRD profiles of the $CFGB_1$ alloy across various temperatures. **e**, Lattice thermal expansion measurements of the $CFGB_1$ alloy.

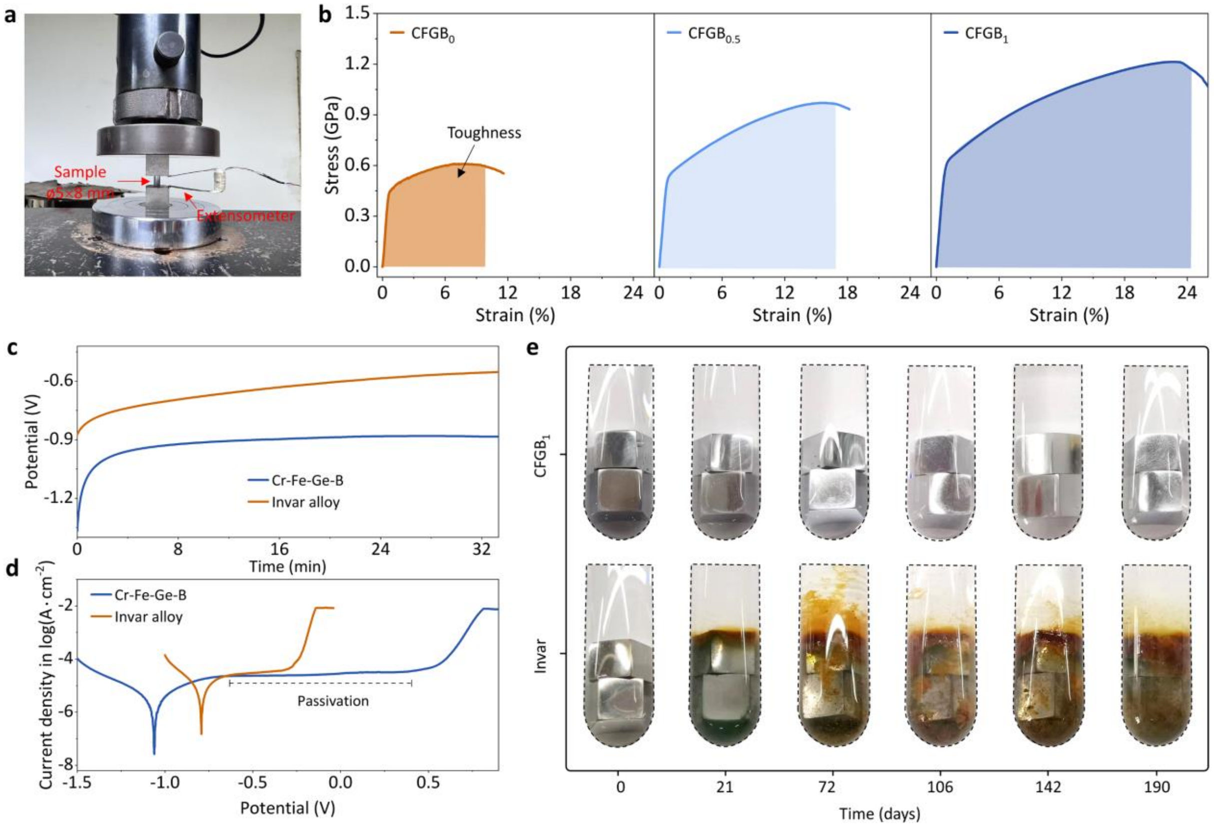

Supplementary Figure 8 | Toughness calculation and corrosion resistance. a, Schematic diagram of the test device. **b**, Toughness integral area calculation. **c**, Electrochemical tests of samples: the open-circuit potential (OCP) curve. **d**, The potentiodynamic polarization (PDP) curve. **e**, images immersed in a 3.5 *wt.* % NaCl solution over various durations.

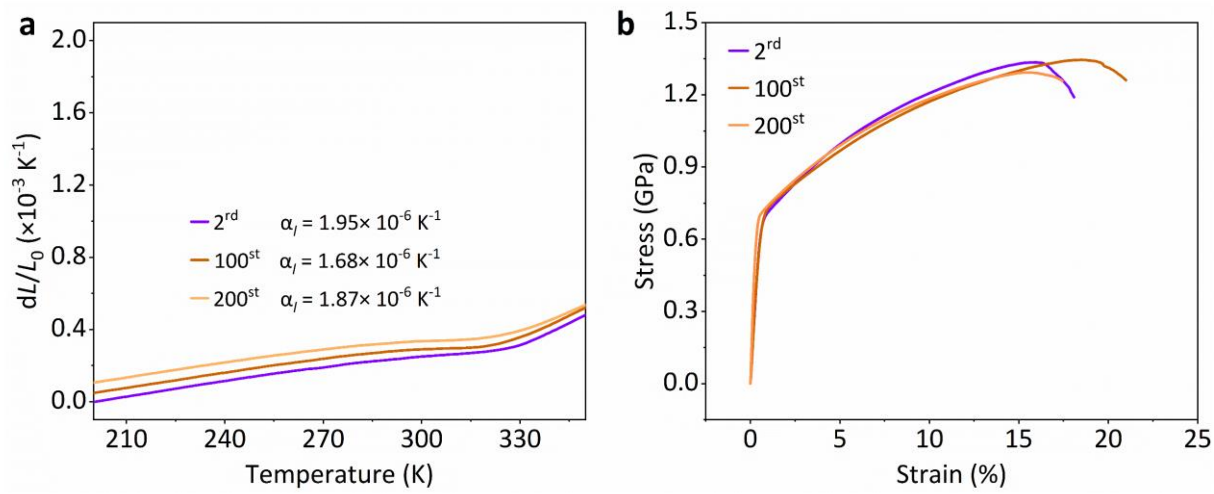

Supplementary Figure 9 | The cyclic thermal shock experiment undergoes a thermal shock from 77 K to 350 K. a, The dilatometer thermal expansions of S-3 alloy in the 2rd, 100th, and 200th cycles. **b,** The compressive stress-strain curves of the S-3 alloy after the 2rd, 100th, and 200th cycles.

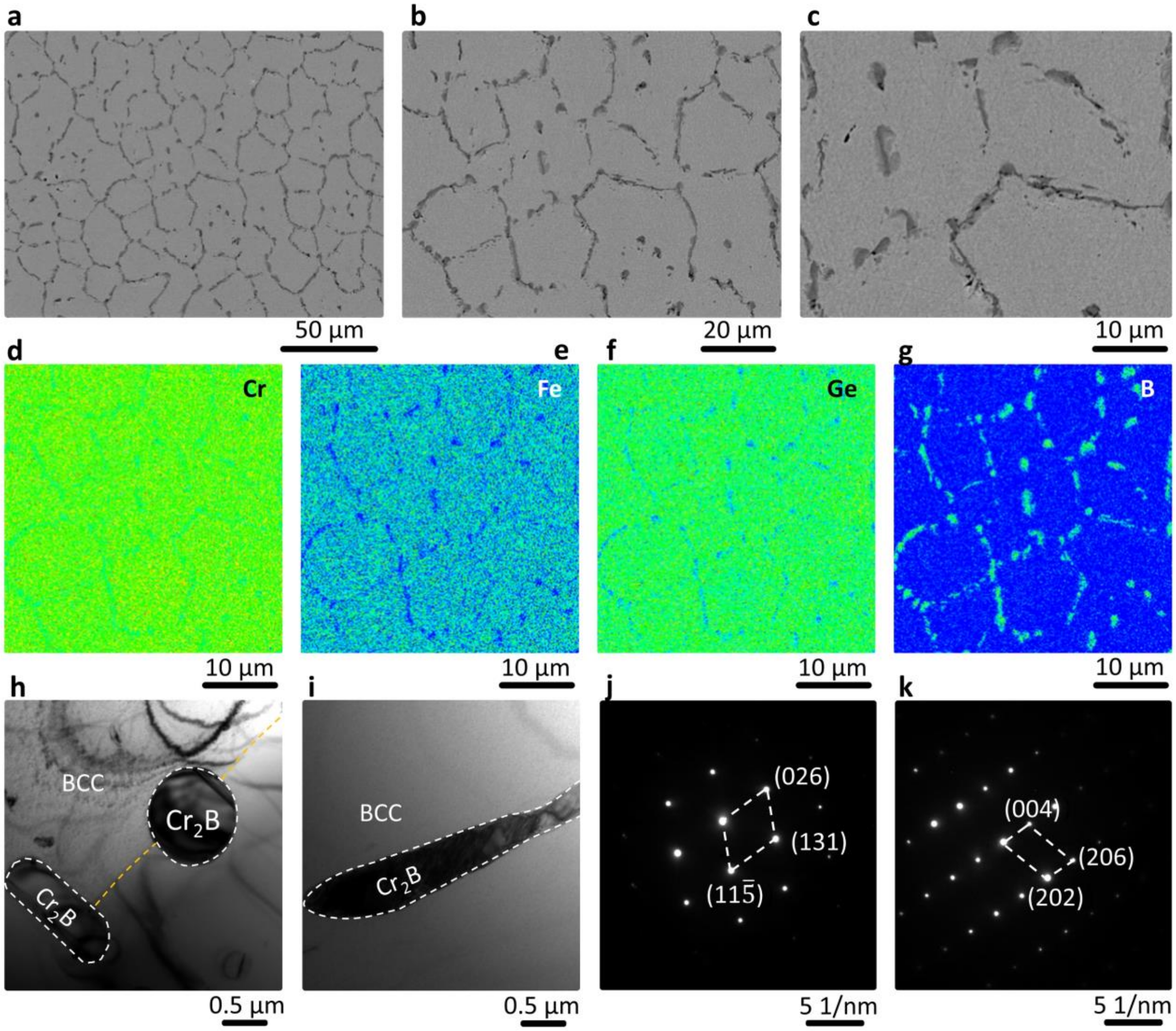

Supplementary Figure 10 | Microstructure of the CFGB$_1$ alloy. a-c, Electro-probe micro-analyzer (EPMA) image of the CFGB$_1$ alloy at different magnifications, showing BCC phase (grey grain), and α phase (black grain). **d-g**, Element mappings of the Cr, Fe, Ge, and B atoms, respectively. **h-k**, Bright-field images **h-i**, and selected area electron diffraction (SAED) of the Cr_2B precipitates (**j-k**).

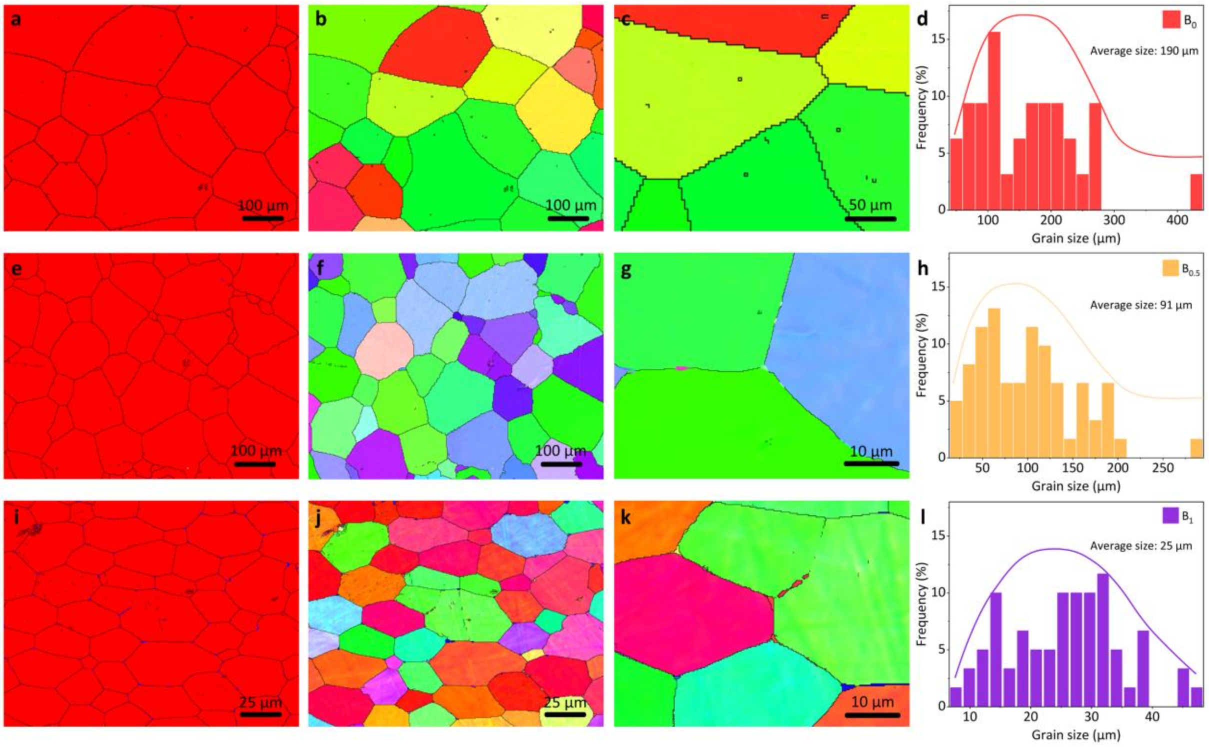

Supplementary Figure 11 | Microstructure evolution of the CFGB1 alloy. a-c, EBSD IPFZ image of CFGB0 alloy at different magnifications. **e-g**, EBSD IPFZ image of CFGB0 alloy at different magnifications. **i-k**, EBSD IPFZ image of CFGB0 alloy at different magnifications. **d**, **h**, **l**, Grain size statistics of different alloy components.

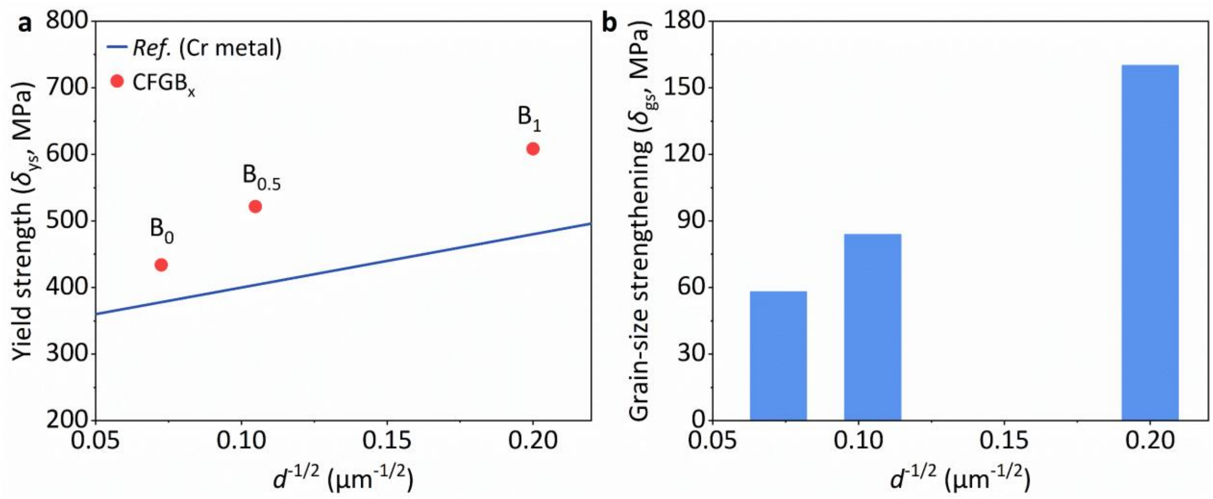

Supplementary Figure 12 | Grain refinement strengthening mechanism of the $CFGB_1$ alloy. **a**, Illustration of the yield strength (σ_y) versus $d^{-1/2}$. **b**, Grain refinement (σ_{gs}) effect of $CFGB_0$, $CFGB_{0.5}$ and $CFGB_1$ alloy, respectively.

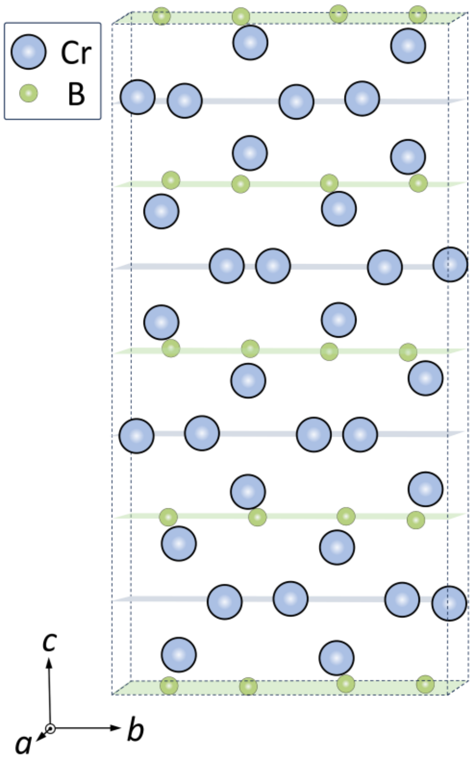

Supplementary Figure 13 | Crystal structure model of the Cr_2B precipitate (Space group: *Fddd*).

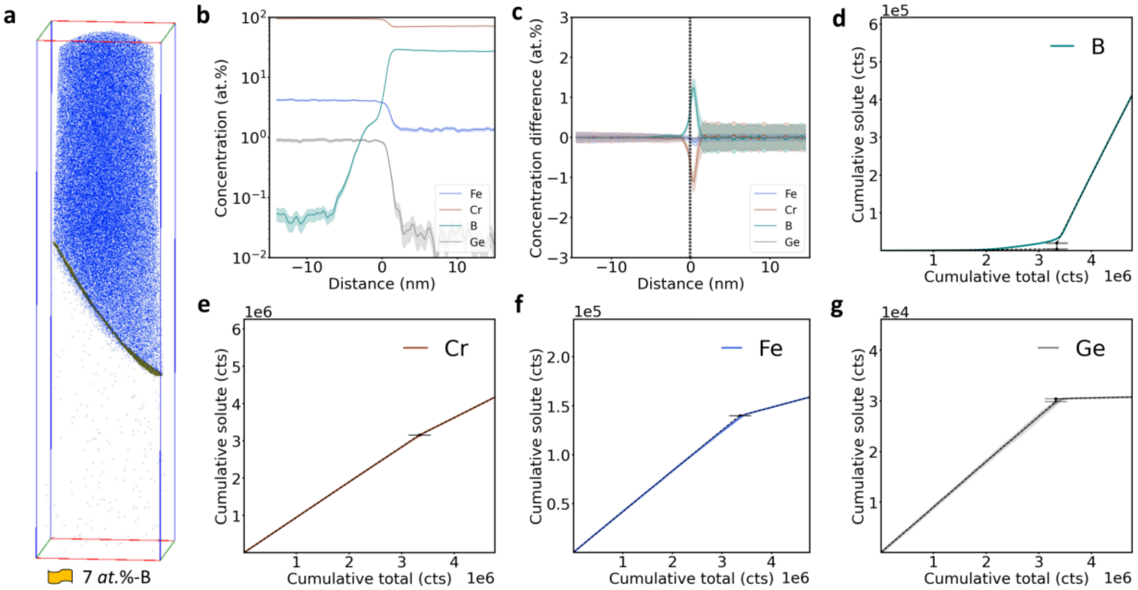

Supplementary Figure 14 | Calculation of Gibbs interfacial excess of boron. a, Boron atom maps reconstructed using 3D-APT of BCC/Cr_2B boundary. **b,** corresponding proximity histograms for the 7-*at.*% B iso-concentration surfaces. **c,** concentration difference profiles indicating the interface location as dashed lines. **d-g,** Cumulative profiles of each element reveal solute excess N_i^{Excess} and its error to calculate the interfacial excess. Error bars represent standard deviation.

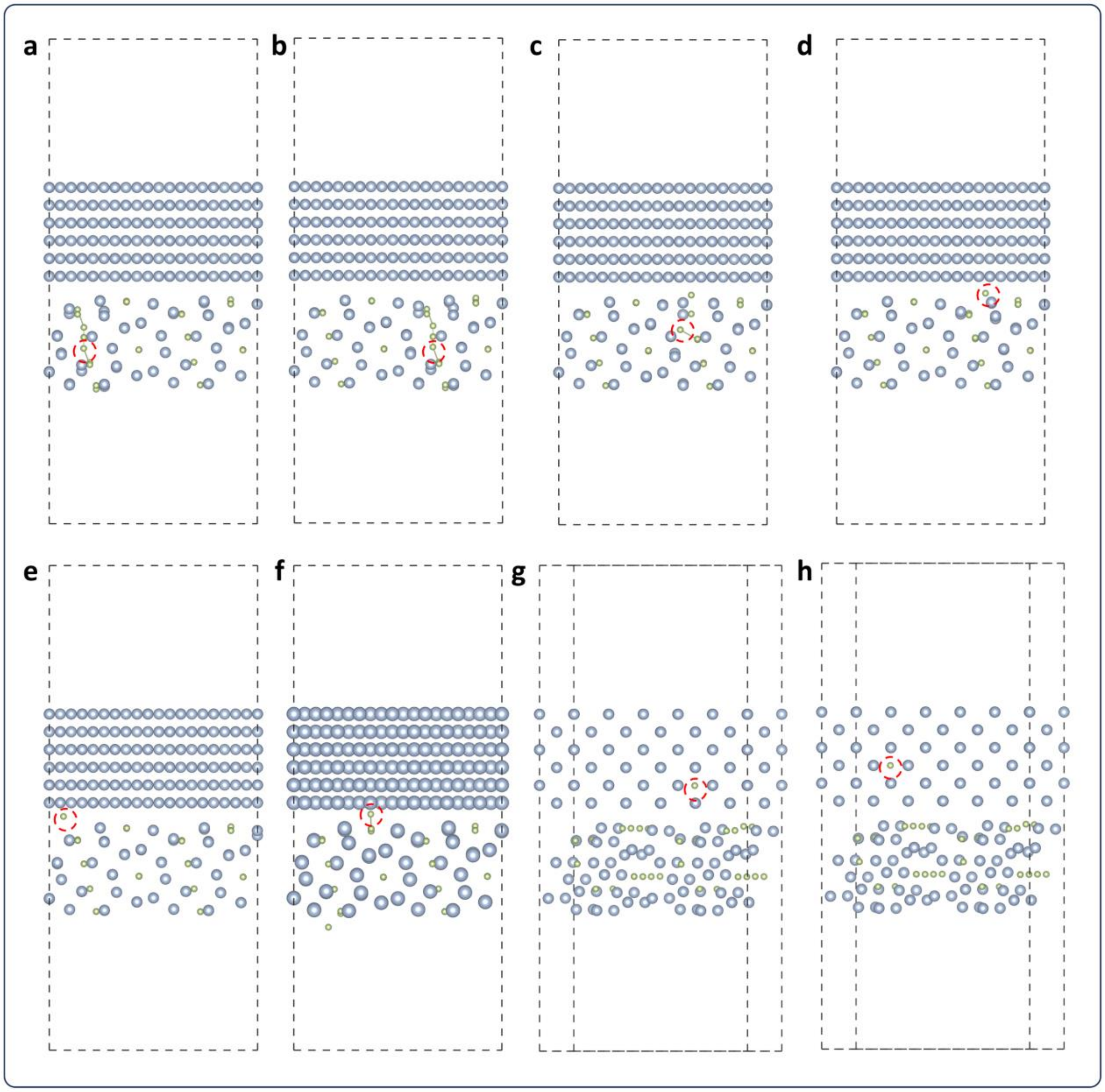

Supplementary Figure 15 | Crystal structure model of the interphase with the boron atoms at various sites. The boron atoms are marked by a red circle.

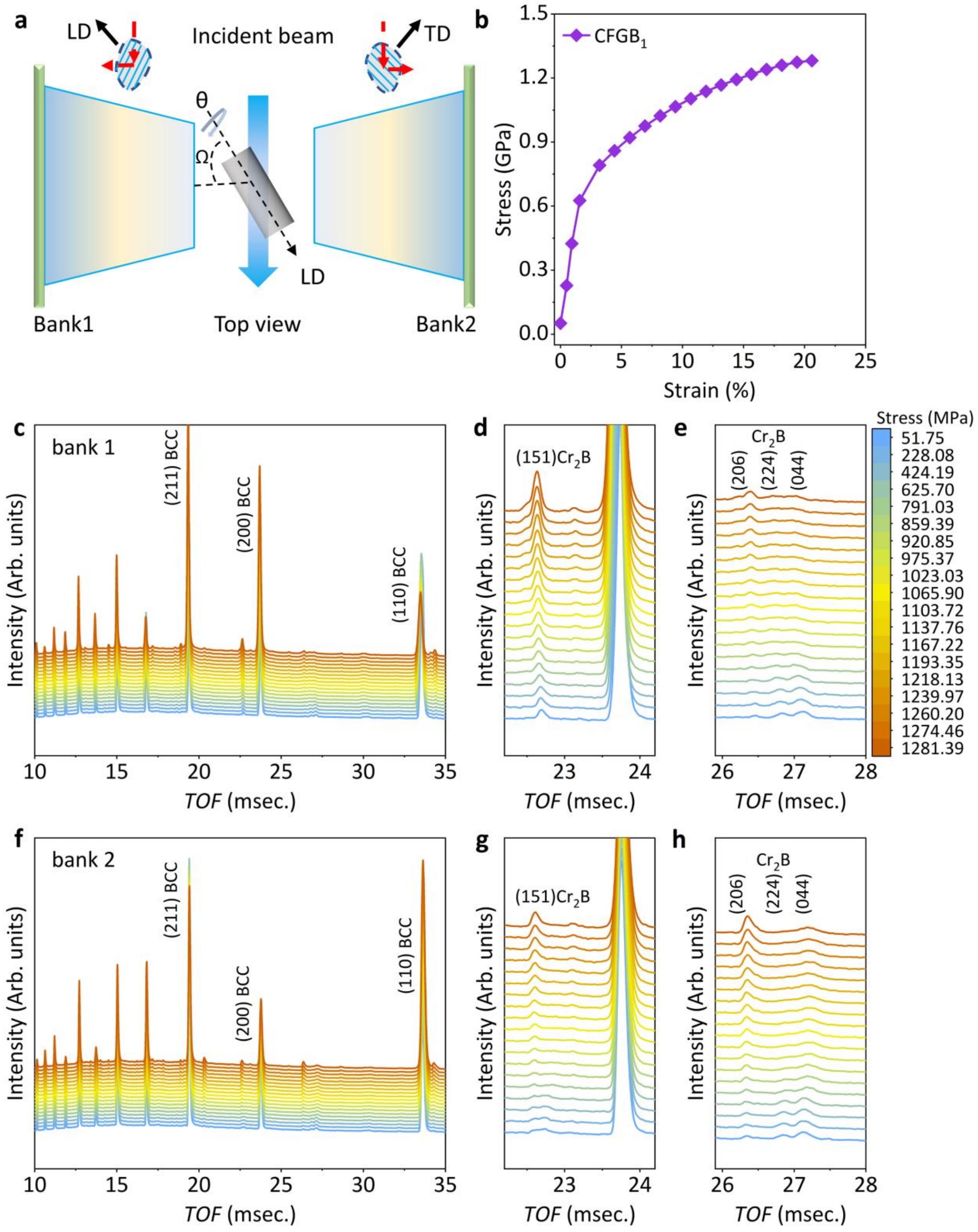

Supplementary Figure 16 | In-situ loading neutron diffraction. **a**, Schematic diagram of the in-situ neutron diffraction experimental set-up from the top view. **b**, Stress-strain curve of $CFGB_1$ determined by in-situ neutron diffraction measurement. **c-h**, Neutron diffraction profiles were collected by bank 1 and bank 2 detectors, respectively.

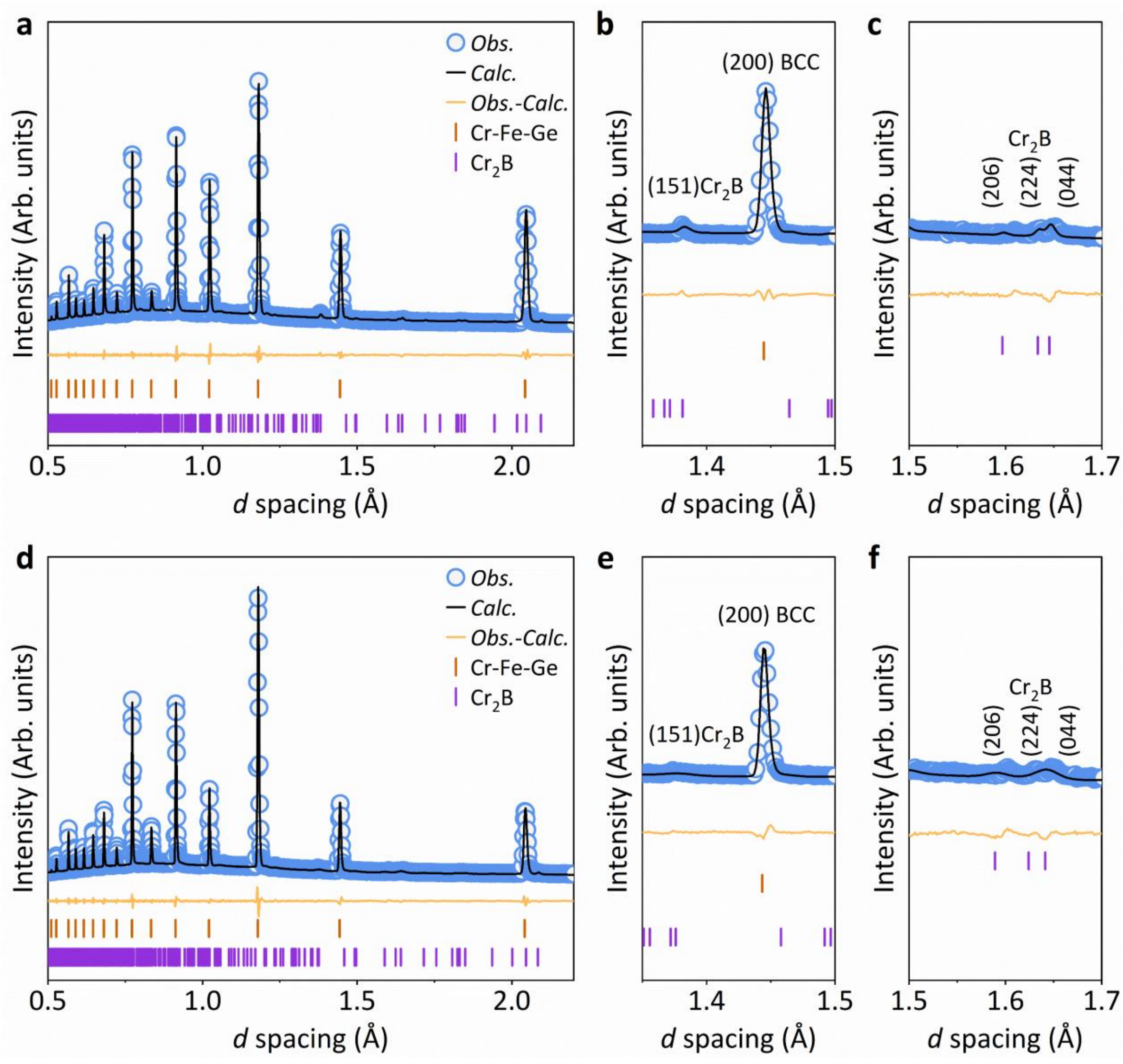

Supplementary Figure 17 | The Rietveld refinement of the CFGB1 alloy. a-c, Profiles before loading direction. **d-f**, Profiles before transverse direction.

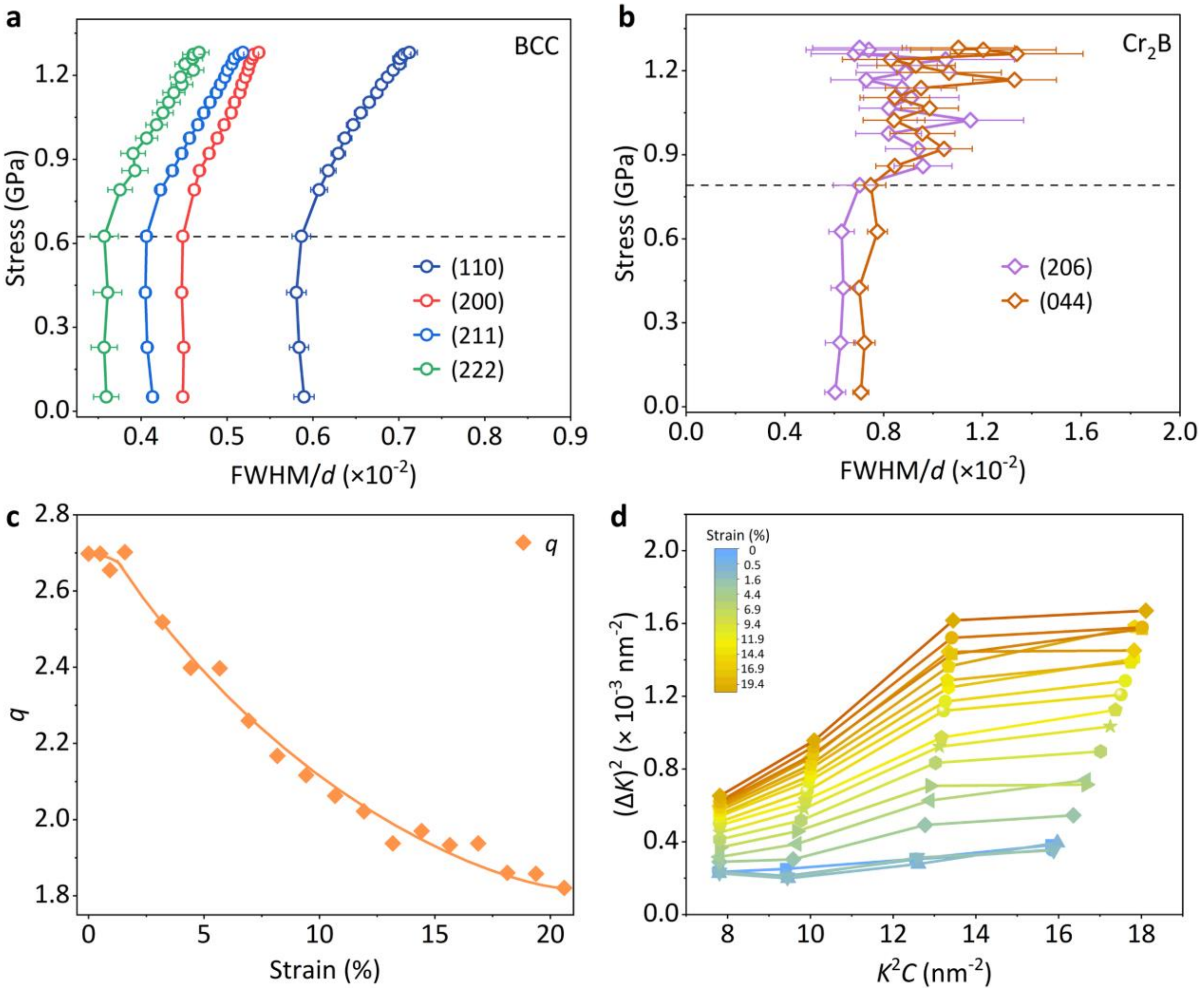

Supplementary Figure 18 | *FWHM/d* and $(\Delta K)^2$ versus $K^2\bar{C}$. a-b, Full width at half maximum (FWHM) of the diffraction peaks for BCC and Cr_2B, respectively. Error bars represent standard deviation. **c**, The curves of q at different strains. **d**, *Modified Williamson-Hall* plot of the $(\Delta K)^2$ versus $K^2\bar{C}$. Error bars represent standard deviation.

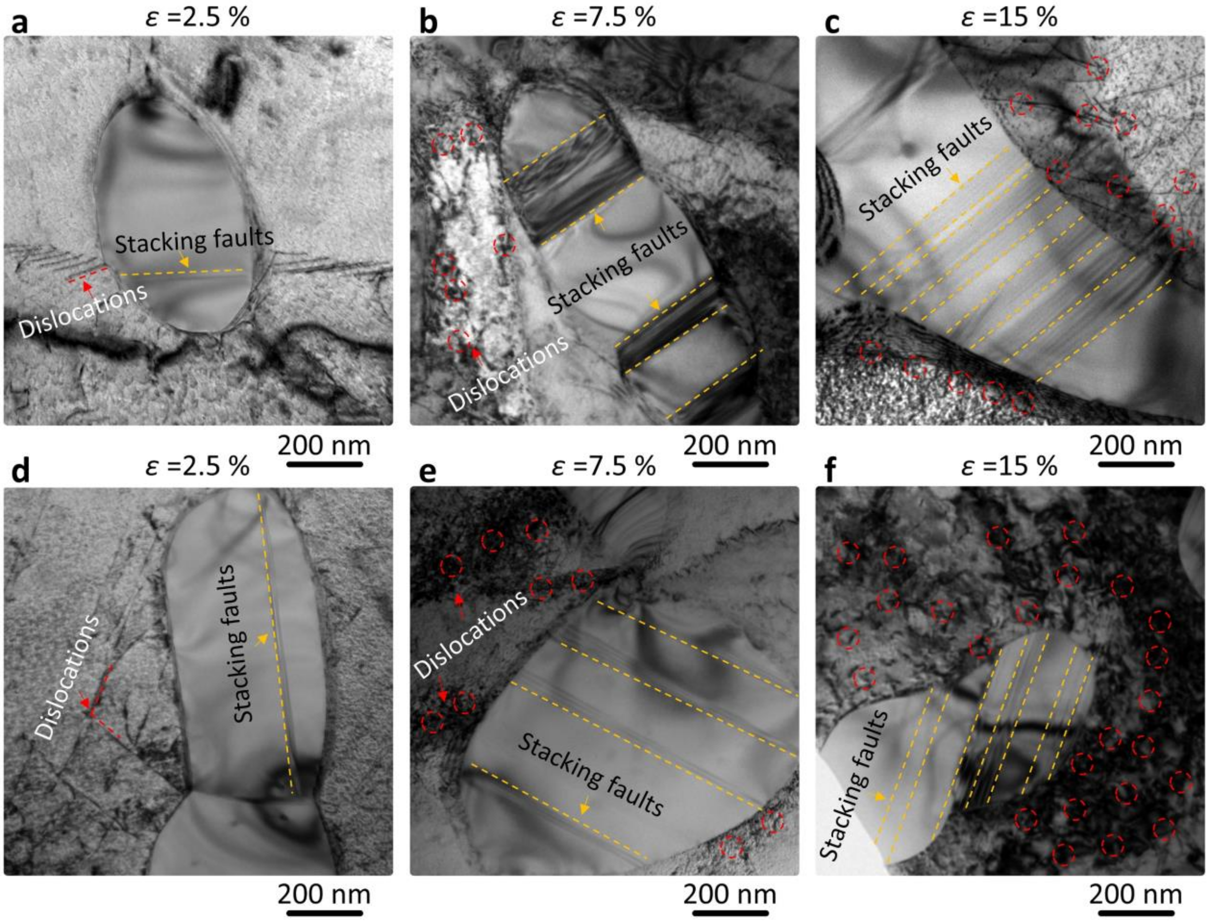

Supplementary Figure 19 | Microstructure evolution of the $CFGB_1$ alloy. a-f, Illustration of the interaction between dislocations and stacking faults at different strains ε = 2.5 (**a, d**), ε = 7.5 % (**b, e**), and ε = 15 % (**c, f**), respectively.

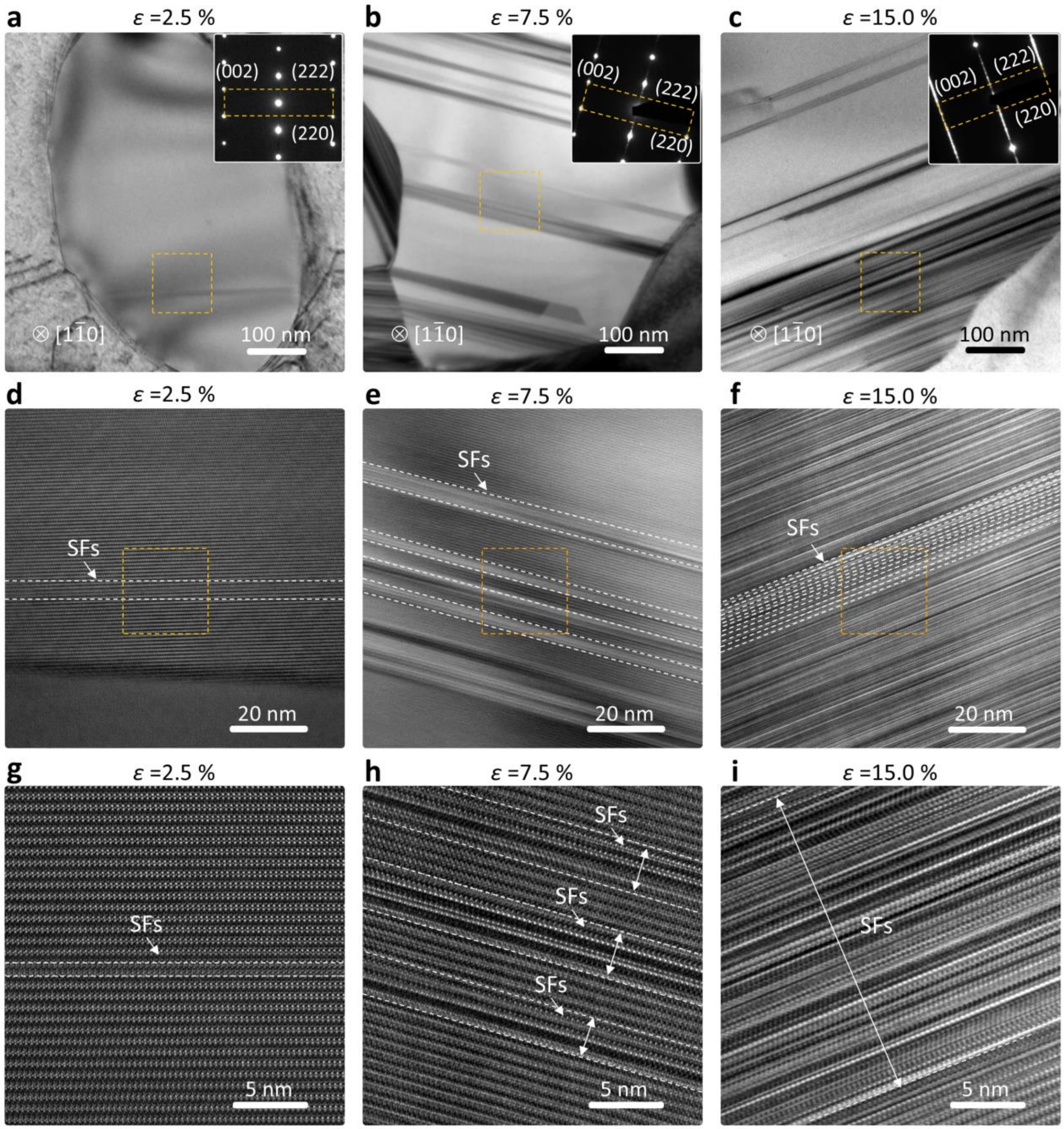

Supplementary Figure 20 | Microstructure evolution of the alloy. a-c, Microstructure of the stacking faults in the $CFGB_1$ alloy along $[1\bar{1}0]_{Cr_2B}$ zone axis at different strain ε = 2.5, ε = 7.5 % and ε = 15 %, respectively. **d-f,** Enlarged HRTEM images marked by yellow rectangular in (**a-c**), respectively. **g-i,** Further enlarged HRTEM images are marked by yellow rectangular in (**d-f**), respectively.

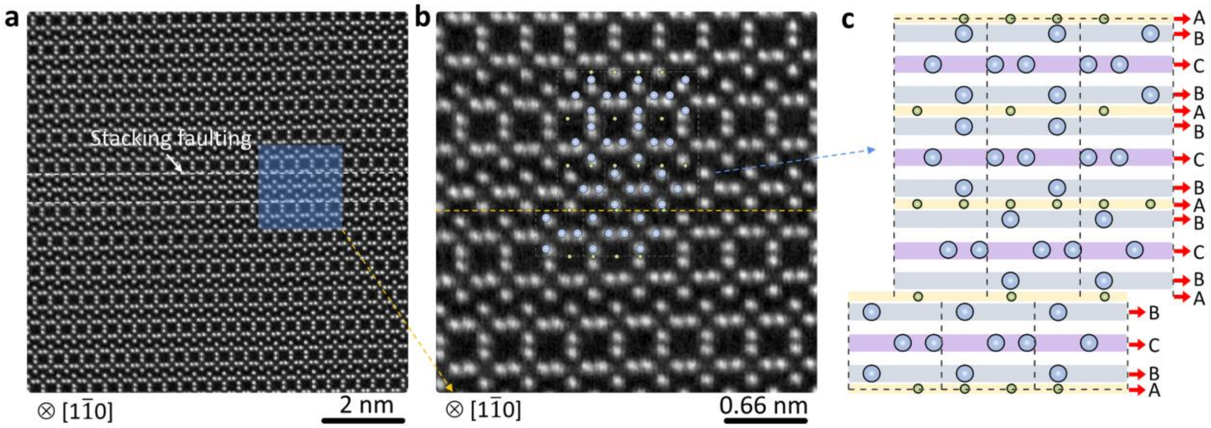

Supplementary Figure 21 | Atomic stacking fault of the Cr_2B phase at ε = 2.5 %. a-b, High-angle annular dark-field scanning TEM (HAADF-STEM) image of the stacking faults along the $[1\bar{1}0]_{Cr_2B}$ zone axis at strain ε = 2.5 %. **c,** The crystal model of the stacking fault.

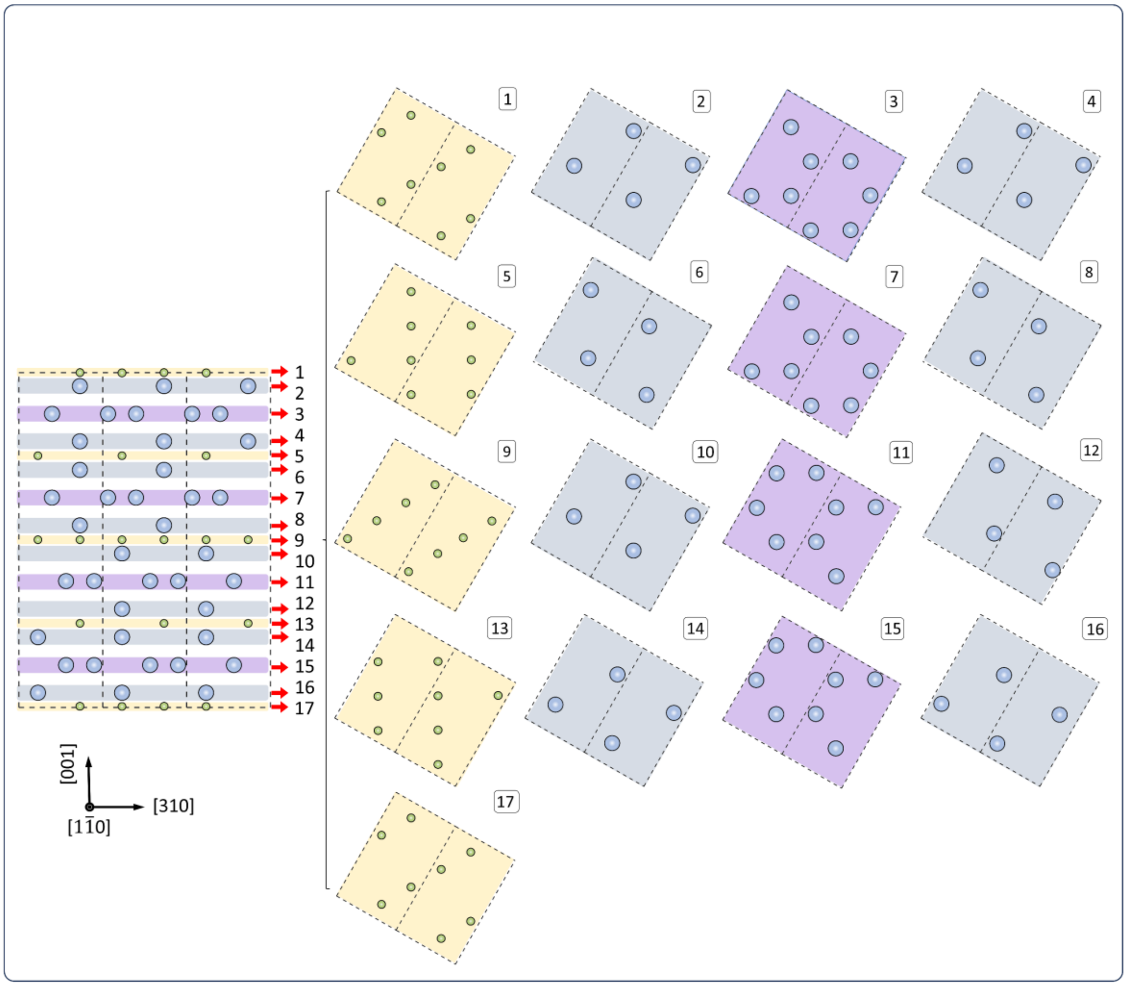

Supplementary Figure 22 | Crystal structure models analysis of the Cr_2B phase along the *c*-axis.

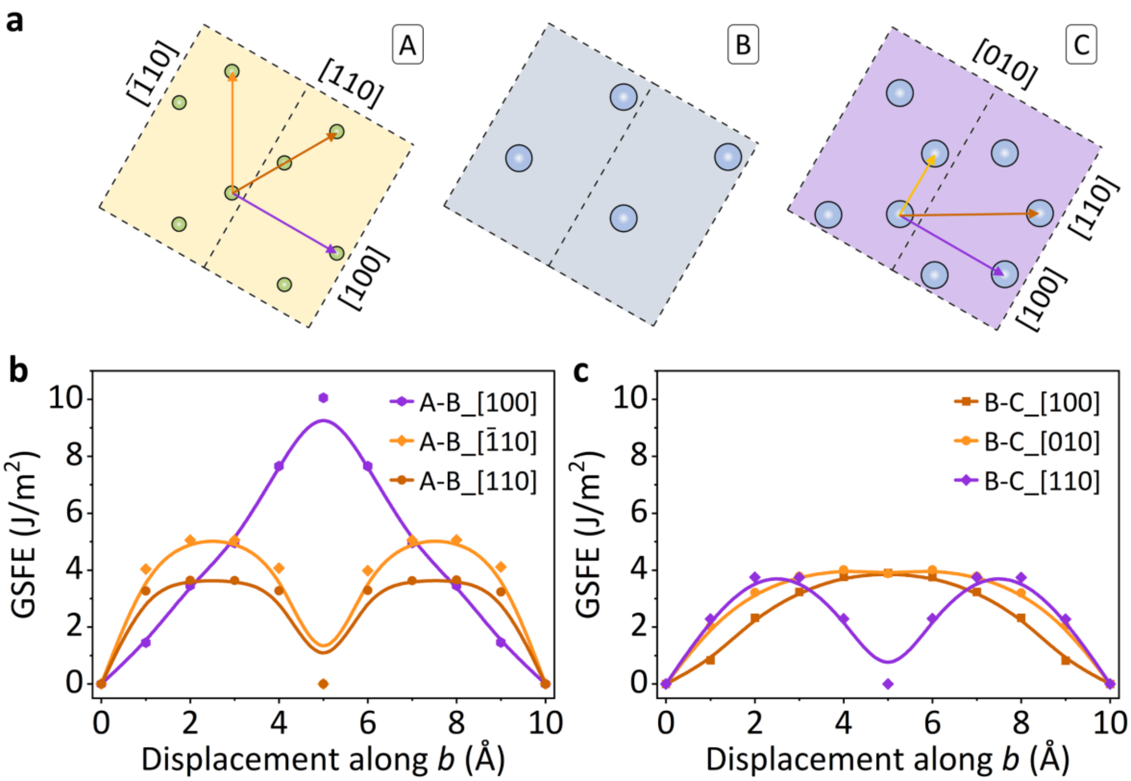

Supplementary Figure 23 | The generalized stacking fault energy (GSFE) along different directions. a, Crystal structure models of the typical A, B, and C layers. **b,** The GSFE of the metal-non-metal layer (**b**) and metal-metal layer (**c**), respectively.

Supplementary References

1. Lohaus S. H., *et al.* A thermodynamic explanation of the Invar effect. *Nat. Phys.* **19**, 1642-1648 (2023).
2. van Schilfgaarde M., Abrikosov I., Johansson B. Origin of the Invar effect in iron-nickel alloys. *Nature* **400**, 46-49 (1999).
3. Cordero Z. C., Knight B. E., Schuh C. A. Six decades of the Hall–Petch effect – a survey of grain-size strengthening studies on pure metals. *International Materials Reviews* **61**, 495-512 (2016).
4. Cao Y., *et al.* Ultrawide temperature range super-Invar behavior of $R_2(Fe,Co)_{17}$ materials (R = Rare Earth). *Phys. Rev. Lett.* **127**, 055501 (2021).
5. Yu C., *et al.* Superior zero thermal expansion dual-phase alloy via boron-migration mediated solid-state reaction. *Nat. Commun.* **14**, 3135 (2023).
6. Li S., Huang R., Zhao Y., Wang W., Han Y., Li L. Zero thermal expansion achieved by an electrolytic hydriding method in $La(Fe,Si)_{13}$ Compounds. *Adv. Funct. Mater.* **27**, 1604195 (2017).
7. Hu J., *et al.* Adjustable magnetic phase transition inducing unusual zero thermal expansion in cubic RCo_2-based intermetallic compounds (R = Rare Earth). *Inorg. Chem.* **58**, 5401-5405 (2019).
8. Song Y., *et al.* Zero thermal expansion in magnetic and metallic $Tb(Co,Fe)_2$ intermetallic compounds. *J. Am. Chem. Soc.* **140**, 602-605 (2018).
9. Li L., *et al.* Good comprehensive performance of Laves phase $Hf_{1-x}Ta_xFe_2$ as negative thermal expansion materials. *Acta Mater.* **161**, 258-265 (2018).
10. Li W., *et al.* A seawater-corrosion-resistant and isotropic zero thermal expansion $(Zr,Ta)(Fe,Co)_2$ Alloy. *Adv. Mater.* **34**, e2109592 (2022).
11. Song Y., *et al.* Opposite thermal expansion in isostructural noncollinear antiferromagnetic compounds of Mn_3A (A = Ge and Sn). *Chem. Mater.* **30**, 6236-6241 (2018).
12. Zhou H., *et al.* Tunable negative thermal expansion in $La(Fe, Si)_{13}$/resin composites with high mechanical property and long-term cycle stability. *Microstructures* **2**, 1-15 (2022).
13. Yu C., *et al.* An isotropic zero thermal expansion alloy with super-high toughness. *Nat. Commun.* **15**, 2252 (2024).
14. Lin K., *et al.* High performance and low thermal expansion in Er-Fe-V-Mo dual-phase alloys. *Acta Mater.* **198**, 271-280 (2020).
15. Pang X., Song Y., Shi N., Xu M., Zhou C., Chen J. Design of zero thermal expansion and high thermal conductivity in machinable *x*LFCS/Cu metal matrix composites. *Compos. Part B-Eng.* **238**, 109883 (2022).
16. Yu C., *et al.* Plastic and low-cost axial zero thermal expansion alloy by a natural dual-phase composite. *Nat. Commun.* **12**, 4701 (2021).
