# Supplementary material for: Atomic faulting drives exceptional toughness in low thermal expansion chromium alloys
Source: Nat Commun. 2026 Feb 6;17:2435. doi: 10.1038/s41467-026-69365-5 (PMC12988168; doi:10.1038/s41467-026-69365-5)
Supplement: Supplementary file 2 — Description of Additional Supplementary Files [file 41467_2026_69365_MOESM2_ESM.pdf]

Description of Additional Supplementary Files

File Name: Supplementary Data 1

Description: This file includes optimized geometric structures of Cr_2B phase obtained through density functional theory (DFT) calculations.
